# Favorable gallbladder cancer mortality-to-incidence ratios of countries with good ranking of world’s health system and high expenditures on health

**DOI:** 10.1186/s12889-019-7160-z

**Published:** 2019-07-31

**Authors:** Chi-Chih Wang, Ming-Chang Tsai, Shao-Chuan Wang, Cheng-Ming Peng, Hsiang-Lin Lee, Hsuan-Yi Chen, Tzu-Wei Yang, Chun-Che Lin, Wen-Wei Sung

**Affiliations:** 1 Institute of Medicine, Chung Shan Medical University, 40201, Taichung, Taiwan; 2 School of Medicine, Chung Shan Medical University, Taichung, 40201 Taiwan; 3 Division of Gastroenterology and Hepatology, Department of Internal Medicine, Chung Shan Medical University Hospital, 40201, Taichung, Taiwan; 4 Department of Urology, Chung Shan Medical University Hospital, 40201, Taichung, Taiwan; 5 Department of Surgery, Chung Shan Medical University Hospital, 40201, Taichung, Taiwan; 6 Institute and Department of Biological Science and Technology, National Chiao Tung University, 30010, Hsinchu, Taiwan

**Keywords:** Gallbladder cancer, Mortality, Incidence, Mortality-to-incidence ratio, Expenditure

## Abstract

**Background:**

The mortality-to-incidence ratio (MIR) is a marker that reflects the clinical outcome of cancer treatment. MIR as a prognostic marker is more accessible when compared with long-term follow-up survival surveys. Theoretically, countries with good health care systems would have favorable outcomes for cancer; however, no report has yet demonstrated an association between gallbladder cancer MIR and the World’s Health System ranking.

**Methods:**

We used linear regression to analyze the correlation of MIRs with the World Health Organization (WHO) rankings and total expenditures on health/gross domestic product (e/GDP) in 57 countries selected according to the data quality.

**Results:**

The results showed high crude rates of incidence/mortality but low MIR in more developed regions. Among continents, Europe had the highest crude rates of incidence/mortality, whereas the highest age-standardized rates (ASR) of incidence/mortality were in Asia. The MIR was lowest in North America and highest in Africa (0.40 and 1.00, respectively). Furthermore, favorable MIRs were correlated with good WHO rankings and high e/GDP (*p* = 0.01 and *p* = 0.030, respectively).

**Conclusions:**

The MIR variation for gallbladder cancer is therefore associated with the ranking of the health system and the expenditure on health.

**Electronic supplementary material:**

The online version of this article (10.1186/s12889-019-7160-z) contains supplementary material, which is available to authorized users.

## Background

Gallbladder cancer (GBC) and extra-hepatic duct cholangiocarcinoma are rare diseases with age standardized incidence rates of around 2 to 3 per 100,000 populations in both gender separately worldwide [1, 2]. GBC is a highly fatal malignancy, with a 5-year survival rate around 13%, and the only effective treatment is early diagnosis [3, 4]. GBC has a prominent geographic variability associated with the prevalence of risk factors [5] such as cholelithiasis [6]. GBC has a higher incidence in Latin America, the Caribbean, and Asia, according to previous studies [5]. GBC has other known characteristics apart from this geographic issue, such as age [7], race, and gender [8], suggesting that the age-standardized rate (ASR) is more reliable than the crude rate as a method for disease evaluation of GBC.

The known risk factors of GBC are gallbladder disease (including gallstones [6], porcelain gallbladder [9], and gallbladder polyps [10]); chronic inflammation of the bile duct (e.g., primary sclerosing cholangitis [11], choledochal cysts, and abnormal pancreaticobiliary duct junctions [12]); and chronic bacterial infections (e.g., *Salmonella* [13] and *Helicobacter* infections [14]) [15]. In terms of these risk factors, congenital abnormalities and chronic bacterial infections are common mainly in low socioeconomic areas, whereas high-fat diets and Caucasian ethnicity [16] increase the possibility of GBC in high socioeconomic countries.

The only treatment that provides a good outcome for GBC is early curative resection. The 5-year mortality rate is around 50% in cases with peri-muscular connective tissue involvement [17], regardless of the treatment choice of surgical resection or adjuvant chemotherapy. The poor response of current chemotherapy regimens means that once GBC invades beyond the gallbladder, the outcome becomes poor and the median survival is only around 3 years [4, 18]. Chemoradiotherapy is another approach for treatment of systemically spreading GBCs, but no randomized trials have yet directly compared the effectiveness of chemotherapy alone versus concomitant chemoradiotherapy [19].

Technical and equipment improvements suggest that health care systems may be able to improve early lesion detection. Better socioeconomic conditions can prevent delays in surgical cholecystectomy, thereby avoiding the extra-gallbladder spread of GBC. We considered that the mortality-to-incidence (MIR) ratio for GBC would be low in a country with a good health care system, as a similar concept has recently been confirmed for prostate and colon cancers [20–23]. The aim of the present study was to clarify the association between World Health Organization (WHO) ranking, geographic region, total expenditure on health/gross domestic product (GDP; e/GDP), and the ASR of GBC incidence and mortality. Our results provide an overview of the MIR and health disparities worldwide for GBC.

## Methods

The data acquisition protocol was described previously [20]. In brief, the cancer incidence and mortality data were obtained from the GLOBOCAN 2012 database, which presented estimates for 2012. The crude rate and ASR are multiplied by 100,000 (cases per 100,000 populations). The database is maintained by the International Agency for Research on Cancer (http://gco.iarc.fr/). The WHO rankings are the World Health Organization’s ranking of the health systems based on an index of factors including health, responsiveness, and fair financial contribution. The health expenditure and life expectancies were obtained from the World Health Statistics 2015 of WHO.

The GLOBOCAN 2012 database contains information for 184 countries. We excluded countries that lacked WHO ranking data (22 countries) or that had a low availability level of data (i.e., a ranking of E to G for incidence or a ranking of 4 to 6 for mortality; 105 countries). Ultimately, 57 countries were used for our analyses. The MIR was defined previously as the ratio of the crude rate of mortality to the incidence [23].

The method used for statistical analysis was described previously [20]. We evaluated the association between the MIRs and variants via linear regression using SPSS statistical software version 15.0 (SPSS, Inc., Chicago, IL). *P* values < 0.05 were considered statistically significant. Scatter plots were produced using Microsoft Excel 2010.

## Results

### The distribution of incidence and mortality numbers/rates in gallbladder cancer according to regions

The incidence/mortality numbers, crude rates, ASR, and MIRs are listed in Table 1. The survey included 178,101 incidences and 142,823 mortalities worldwide. The more developed regions had higher crude rates of incidence/mortality, but favorable ASR and MIRs, when compared with the less developed regions. In the categories of WHO regions, the WHO Western Pacific region had the highest number, crude rates, and ASR of incidence/mortality. However, when we grouped the countries via continent, the highest crude rates of incidence/mortality were in Europe and the highest ASR of incidence/mortality was in Asia. In both categories, Africa had the highest MIR and the WHO Americas region and North America had the lowest MIR.

**Table 1:** Summary of the case number, rates and mortality-to-incidence ratio of the incidence and mortality according to regions in gallbladder cancer

Region	Number		Crude rate		Age-standardized rate		Mortality-to-incidence ratio^a^
	Incidence	Mortality	Incidence	Mortality	Incidence	Mortality	
World	178,101	142,823	2.5	2.0	2.2	1.7	0.80
Development							
More developed regions	62,535	44,843	5.0	3.6	2.1	1.4	0.72
Less developed regions	115,566	97,980	2.0	1.7	2.2	1.8	0.85
WHO region categories							
WHO Africa region	2762	2597	0.3	0.3	0.6	0.6	1.00
WHO Americas region	25,857	17,240	2.7	1.8	2.0	1.3	0.67
WHO East Mediterranean region	5509	5133	0.9	0.8	1.3	1.2	0.89
WHO Europe region	31,409	22,352	3.5	2.5	1.7	1.2	0.71
WHO South-East Asia region	31,003	27,002	1.7	1.5	1.9	1.7	0.88
WHO Western Pacific region	81,549	68,486	4.4	3.7	3.1	2.5	0.84
Continent							
Africa	4559	4271	0.4	0.4	0.7	0.7	1.00
Latin America and Caribbean	15,278	12,935	2.5	2.1	2.4	2.0	0.84
Northern America	10,579	4305	3.0	1.2	1.6	0.6	0.40
Asia	117,076	100,020	2.8	2.4	2.6	2.2	0.86
Europe	29,744	20,887	4.0	2.8	1.8	1.2	0.70
Oceania	865	405	2.3	1.1	1.5	0.7	0.48

^a^the percentage in the ratio of the crude rate of mortalities and the crude rate of incidences

### The World Health Organization ranking and total health expenditure are correlated with the mortality-to-incidence ratios in gallbladder cancer

The data for 57 selected countries are summarized in Table 2. The mean e/GDP was 8.0%, with a standard deviation of 2.6 (ranging from 4.0% [Malaysia and Fiji] to 17.0% [United States of America]). Among the 57 countries, Japan had the highest incidence and mortality number for GBC. For both the incidence and mortality rates, Japan had the highest incidence and mortality crude rates and Chile had the highest incidence and mortality ASR. Five countries had MIR values greater than or equal to 1.00, including Sweden (1.23), Estonia (1.12), Egypt (1.00), the Republic of South Africa (1.00), and Oman (1.00).

**Table 2:** Summary of World Health Organization rankings, total expenditure on health/GDP, life expectancy, gallbladder cancer incidence, mortality, and mortality-to-incidence ratio of selected countries

Country	Ranking	Total expenditure on health/GDP (%)	Life expectancy	Number		Crude rate		Age-standardized rate		Mortality-to-incidence ratio^a^
				Incidence	Mortality	Incidence	Mortality	Incidence	Mortality	
France	1	11.6	82	2512	1132	4.0	1.8	1.6	0.6	0.45
Italy	2	9.2	83	3945	3364	6.5	5.5	2.3	1.8	0.85
Malta	5	8.7	81	15	5	3.6	1.2	1.8	0.6	0.33
Singapore	6	4.2	83	138	75	2.6	1.4	1.7	0.9	0.54
Spain	7	9.3	83	2002	1174	4.3	2.5	1.7	0.9	0.58
Oman	8	2.7	76	12	12	0.4	0.4	0.7	0.7	1.00
Austria	9	11.1	81	359	224	4.3	2.7	1.7	1.0	0.63
Japan	10	10.3	84	21,417	19,309	16.9	15.3	4.7	3.9	0.91
Norway	11	9.3	82	160	39	3.2	0.8	1.6	0.3	0.25
Portugal	12	9.9	81	496	303	4.6	2.8	2.1	1.1	0.61
Iceland	15	9.0	82	7	3	2.1	0.9	1.2	0.4	0.43
Luxembourg	16	7.2	82	5	0	1.0	0.0	0.6	0.0	0.00
Netherlands	17	12.7	81	635	353	3.8	2.1	1.8	1.0	0.55
United Kingdom	18	9.3	81	750	687	1.2	1.1	0.5	0.4	0.92
Ireland	19	8.9	81	152	44	3.3	1.0	2.0	0.5	0.30
Switzerland	20	11.4	83	331	208	4.3	2.7	1.8	1.1	0.63
Belgium	21	10.9	80	370	193	3.4	1.8	1.5	0.6	0.53
Colombia	22	6.8	78	1227	909	2.6	1.9	2.8	2.1	0.73
Sweden	23	9.6	82	368	456	3.9	4.8	1.7	1.9	1.23
Cyprus	24	7.3	82	28	13	2.5	1.2	1.4	0.7	0.48
Germany	25	11.3	81	5340	2913	6.5	3.6	2.3	1.2	0.55
Israel	28	7.4	82	177	73	2.3	0.9	1.5	0.6	0.39
Canada	30	10.9	82	1148	460	3.3	1.3	1.6	0.6	0.39
Finland	31	9.1	81	256	203	4.7	3.8	1.8	1.4	0.81
Australia	32	8.9	83	680	251	3.0	1.1	1.5	0.5	0.37
Chile	33	7.3	80	2280	1879	13.1	10.8	9.7	7.8	0.82
Denmark	34	11.0	80	255	128	4.6	2.3	2.1	1.0	0.50
Costa Rica	36	10.1	79	103	62	2.1	1.3	2.0	1.3	0.62
United States of America	37	17.0	79	9431	3845	3.0	1.2	1.6	0.6	0.40
Slovenia	38	9.4	80	198	147	9.7	7.2	3.9	2.8	0.74
Cuba	39	8.6	78	254	168	2.3	1.5	1.3	0.8	0.65
New Zealand	41	10.2	82	115	91	2.6	2.0	1.4	1.0	0.77
Bahrain	46	4.4	77	6	1	0.4	0.1	0.8	0.2	0.25
Thailand	47	4.5	75	2794	2381	4.0	3.4	3.0	2.5	0.85
Czech Republic	48	7.5	78	966	760	9.1	7.2	4.1	3.1	0.79
Malaysia	49	4.0	74	248	150	0.8	0.5	1.0	0.6	0.63
Poland	50	6.8	77	2296	1894	6.0	4.9	2.9	2.4	0.82
Jamaica	53	5.6	74	29	26	1.1	0.9	0.9	0.8	0.82
Korea, Republic of	58	7.6	82	5228	4176	10.8	8.6	6.5	4.8	0.80
Philippines	60	4.4	69	342	256	0.4	0.3	0.5	0.4	0.75
Slovakia	62	8.1	76	394	296	7.2	5.4	4.0	3.0	0.75
Egypt	63	4.9	71	789	727	0.9	0.9	1.1	1.1	1.00
Uruguay	65	8.6	77	203	180	6.0	5.3	3.3	2.9	0.88
Trinidad and Tobago	67	5.5	71	17	16	1.3	1.2	1.1	1.1	0.92
Belarus	72	5.0	72	218	110	2.3	1.2	1.3	0.7	0.52
Lithuania	73	6.7	74	106	74	3.2	2.2	1.5	1.0	0.69
Argentina	75	6.8	76	1882	1466	4.6	3.6	3.3	2.5	0.78
Estonia	77	5.9	77	44	49	3.3	3.7	1.4	1.6	1.12
Ukraine	79	7.5	71	1036	830	2.3	1.8	1.2	1.0	0.78
Mauritius	84	4.8	74	20	18	1.5	1.4	1.3	1.2	0.93
Fiji	96	4.0	70	10	9	1.1	1.0	1.4	1.1	0.91
Bulgaria	102	7.4	75	303	174	4.1	2.4	1.8	1.0	0.59
Latvia	105	5.9	74	60	51	2.7	2.3	1.2	0.9	0.85
Ecuador	111	6.4	76	562	379	3.8	2.5	4.0	2.7	0.66
Brazil	125	9.5	75	4049	3525	2.0	1.8	1.9	1.6	0.90
Russian Federation	130	6.5	69	3411	2834	2.4	2.0	1.3	1.1	0.83
South African Republic	175	8.9	60	219	199	0.4	0.4	0.6	0.5	1.00

^a^the percentage in the ratio of the crude rate of mortalities and the crude rate of incidences

**Fig. 1:**
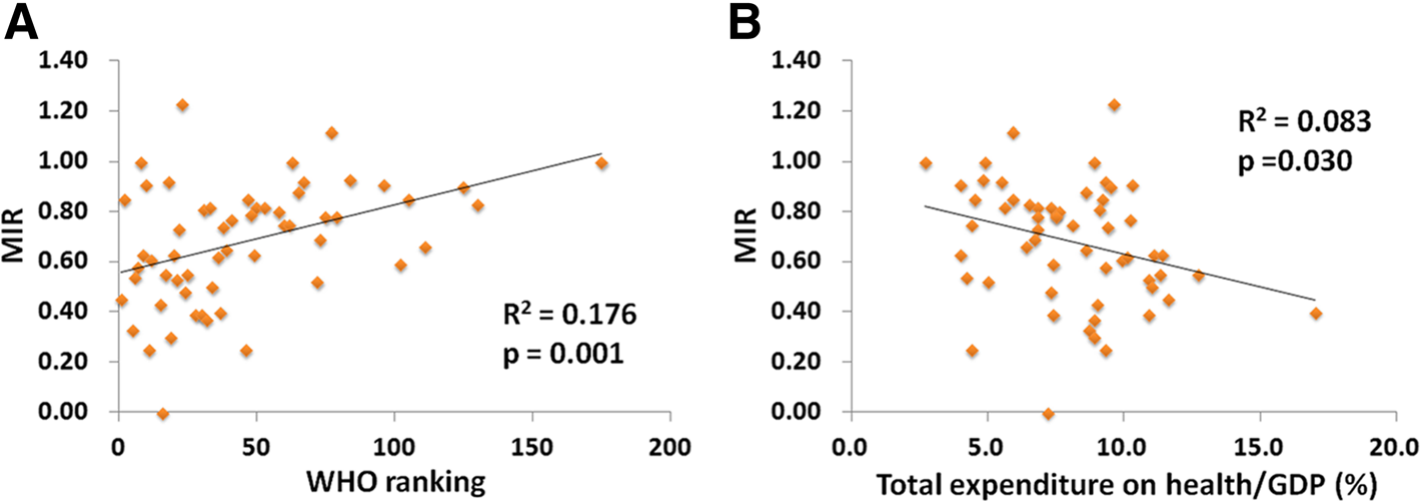
The (**a**) World Health Organization rankings and (**b**) total expenditures on health/GDP are significantly associated with the MIR in gallbladder cancer

The association between crude rate/age-standardized rate of incidence/mortality and the WHO ranking or e/GDP is illustrated in Additional file 1: Figure S1 and S2. No significant association was noted, except between the e/GDP and the crude rate of incidence (*p* = 0.031, Additional file 1: Figure S2A). The favorable MIRs of 57 countries were significantly associated with good WHO ranking and high e/GDP (R^2^ = 0.176, *p* = 0.001; R^2^ = 0.083, *p* = 0.030, respectively, Fig. 1).

## Discussion

In this study, we analyzed the correlation of incidence, mortality, and MIRs for GBC with WHO rankings and total expenditures on health/GDP. A correlation between MIR and health care disparities was confirmed previously for many cancers [20, 23]. Our analysis showed that the more developed regions have higher crude rates of incidence and mortality but favorable ASR and MIRs when compared with the less developed regions. The developed regions and countries have greater numbers of elderly people and older age is one important risk factor of GBC; consequently, the crude rates of incidence and mortality were higher in the more developed regions, but the condition was reversed for ASR and MIRs. The geographic continent analysis revealed that the WHO Western Pacific region and Asia had the highest ASR of incidence and mortality for GBC and these results were similar to those of previous studies of geographic regions [5, 24] published decades ago. Both the WHO America region and North America showed a median ASR of incidence and the lowest age-standardized mortality and MIRs. These results imply an importance of economic level and e/GDP in GBC prognosis.

The best treatment choice is early diagnosis, so screening programs and high probability population for GBC have been established in some studies [25, 26]. The availability of precision instruments and experienced physicians are known key factors for the early diagnosis of GBC. Africa had the lowest crude rates and ASR of incidence/mortality, but the highest MIR around the world.

The WHO ranking and e/GDP showed no significant correlations with the crude rate and ASR of incidence/mortality, except for the crude rate of incidence. We found an association between a higher e/GDP and a higher crude rate of incidence for GBC in our analysis. We believe that the higher e/GDP countries have better or more frequent screening programs, which lead to more GBC diagnoses. Furthermore, the favorable MIRs of 57 countries are significantly associated with good WHO ranking and high e/GDP (R^2^ = 0.176, *p* = 0.001; R^2^ = 0.083, *p* = 0.030, respectively, Fig. 1).

This study has some limitations. First, many countries, and especially those in the least developed areas in the world, do not participate in the WHO, and this may influence the impact of total e/GDP on GBC incidence. Second, the ethnicity, geographic region, and national health insurance issues (especially for e/GDP) could not be fully analyzed in our study, and these issues may add some bias to our study. Third, the use of MIR for predicting disease outcome has many limitations, since MIR was calculated from the cross-sectional data of mortality and incidence for a certain period causing different patients calculated in the incidence and mortality. MIR would not substitute for prognostic data from long-term follow up or from a cohort study. Forth, we excluded countries with relatively poor or unknown data quality which changes the distribution of countries according to the regions or continents. We analyzed the main results without country selection, the conclusion remains unchanged. The favorable MIRs of all countries were significantly associated with good WHO ranking and high e/GDP (R^2^ = 0.309, *p* < 0.001; R^2^ = 0.118, p < 0.001, respectively, Additional file 1: Figure S3). Other limitations include the lack of detailed information about the disease clinical parameters, health care facilities or policies, socioeconomic determinant, and confounding factors of cancer risks. Despite these limitations, MIR appears to provide more accessible data when compared with long-term follow up survival surveys.

## Conclusions

In conclusion, the MIRs of GBC showed a significant correlation with the WHO ranking and e/GDP in this study. We successfully demonstrated that MIRs could reflect the health care disparities in GBC worldwide and could explain the differences in crude rates and ASR of incidence and mortality between WHO region categories and geographic continents.

## Additional file

Additional file 1: **Figure S1.** The association between the World Health Organization rankings and the crude rates of (A) incidence, and (B) mortality; the ASR of (C) incidence, and (D) mortality. **Figure S2.** The association between the total expenditures on health/GDP and the crude rates of (A) incidence, and (B) mortality; the ASR of (C) incidence, and (D) mortality. **Figure S3.** The (A) World Health Organization rankings (*N* = 142) and (B) total expenditures on health/GDP (*N* = 139) are significantly associated with the MIR in gallbladder cancer under investigation without country selection. (DOCX 739 kb)

## Data Availability

All the data were obtain from global statistic of GLOBOCAN (http://gco.iarc.fr/). The database of GLOBOCAN 2012 is closed while this manuscript was under revision since the updated database is released. The database might be available upon request to the IARC.

## Abbreviations

ASR: age-Standardized rate
e/GDP: Total expenditures on health/gross domestic product
GBC: Gallbladder cancer
GDP: Gross domestic product
MIR: Mortality-to-incidence ratio
WHO: World Health Organization
