# Supplementary material for: Favorable gallbladder cancer mortality-to-incidence ratios of countries with good ranking of world’s health system and high expenditures on health
Source: BMC Public Health. 2019 Jul 31;19:1025. doi: 10.1186/s12889-019-7160-z (PMC6670146; doi:10.1186/s12889-019-7160-z)
Supplement: Supplementary file 1 — Figure S1. The association between the World Health Organization rankings and the crude rates of (A) incidence, and (B) mortality; the ASR of (C) incidence, and (D) mortality. Figure S2. The association between the total expenditures on health/GDP and the crude rates of (A) incidence, and (B) mortality; the ASR of (C) incidence, and (D) mortality. Figure S3. The (A) World Health Organization rankings (N = 142) and (B) total expenditures on health/GDP (N = 139) are significantly associated with the MIR in gallbladder cancer under investigation without country selection. (DOCX 739 kb) [file 12889_2019_7160_MOESM1_ESM.docx]

**Supporting Information**


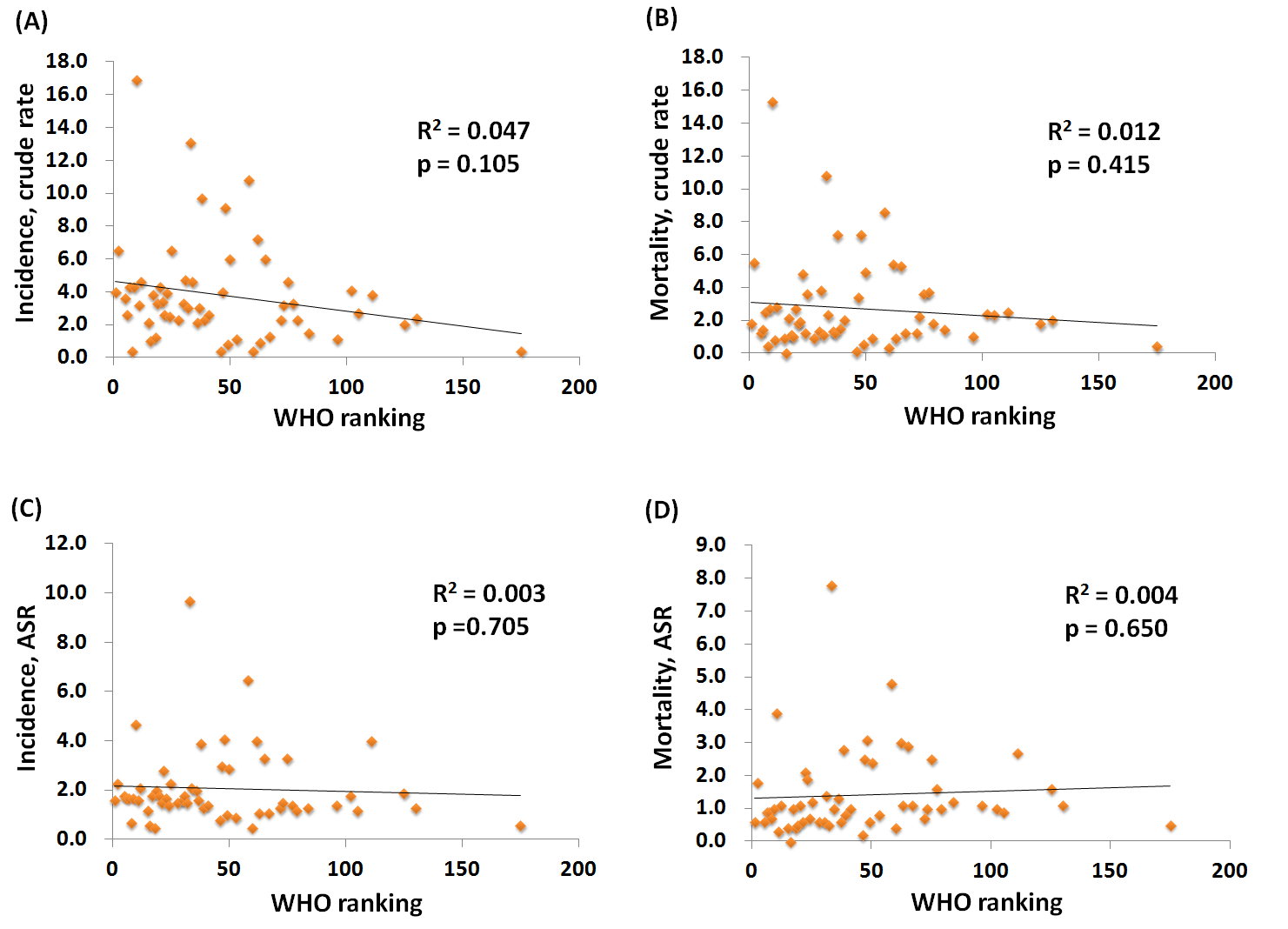
S1 Fig. The association between the World Health Organization rankings and the crude rates of (A) incidence, and (B) mortality; the ASR of (C) incidence, and (D) mortality.


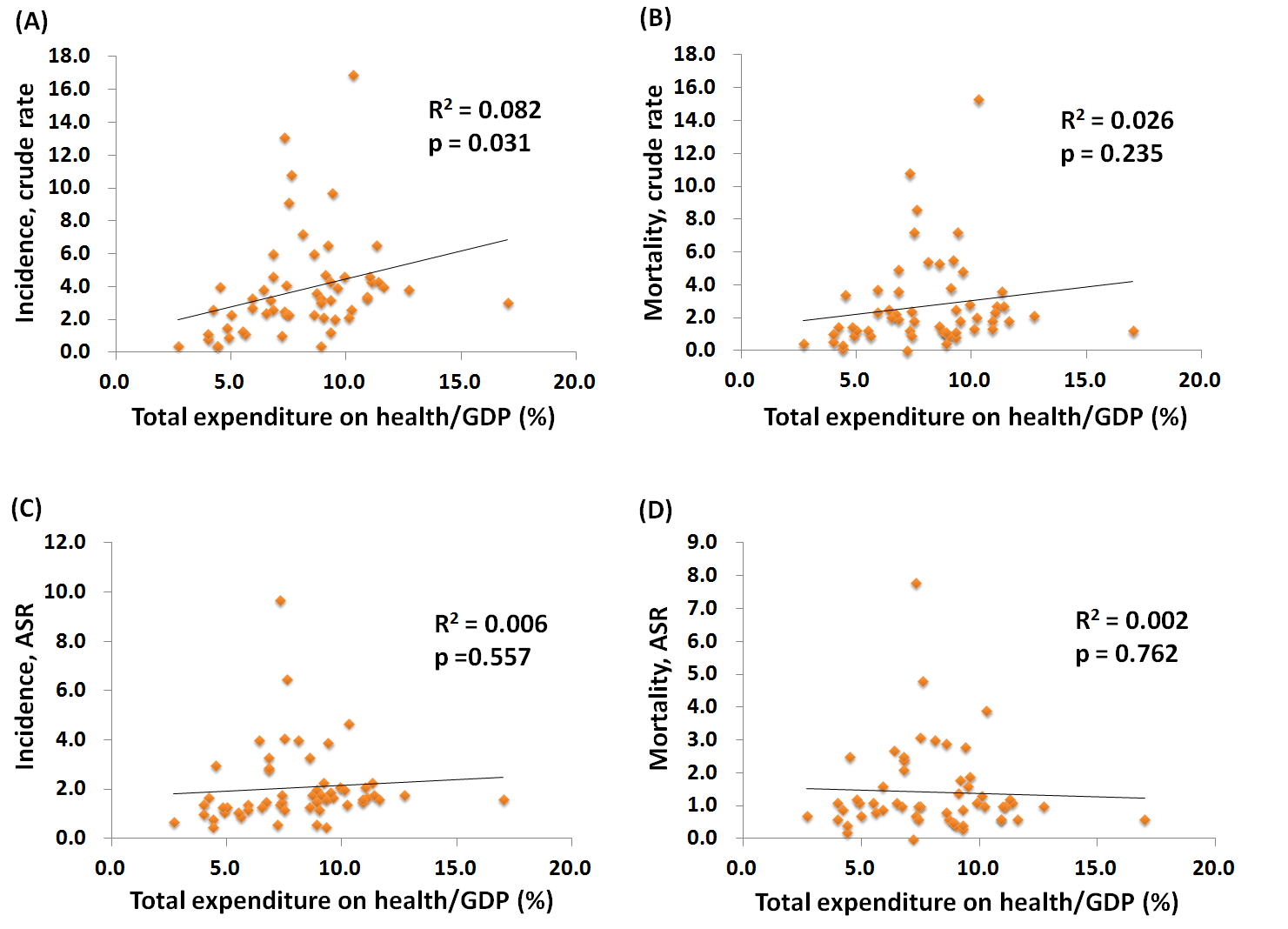


SF2. The association between the total expenditures on health/GDP and the crude rates of (A) incidence, and (B) mortality; the ASR of (C) incidence, and (D) mortality.


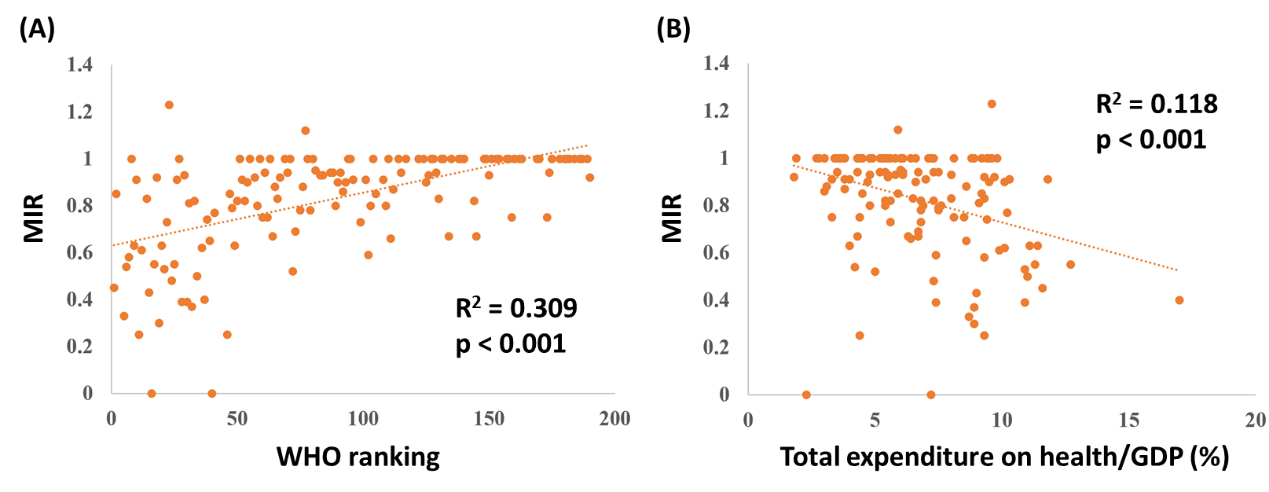


SF3. The (A) World Health Organization rankings (N = 142) and (B) total expenditures on health/GDP (N=139) are significantly associated with the MIR in gallbladder cancer under investigation without country selection.
